# GDF15/GFRAL Pathway as a Metabolic Signature for Cachexia in Patients with Cancer

**DOI:** 10.7150/jca.50376

**Published:** 2021-01-01

**Authors:** Darakhshan Sohail Ahmed, Stéphane Isnard, John Lin, Bertrand Routy, Jean-Pierre Routy

**Affiliations:** 1 Infectious Disease and Immunity in Global Health Program, Research Institute of McGill University Health Centre, Montreal, QC, Canada; 2 Division of Hematology and Chronic Viral Illness Service, McGill University Health Centre, Montreal, QC, Canada; 3 CIHR Canadian HIV Trials Network, Vancouver, BC; 4 Division of Hémato-oncologie, Centre hospitalier de l'Université de Montréal; 5 Centre de recherche du Centre hospitalier de l'Université de Montréal; 6 Division of Hematology, McGill University Health Centre, Montreal, QC, Canada

**Keywords:** GDF15, GFRAL, Inflammation, cachexia, cancer

## Abstract

Cachexia is a metabolic mutiny that directly reduces life expectancy in chronic conditions such as cancer. The underlying mechanisms associated with cachexia involve inflammation, metabolism, and anorexia. Therefore, the need to identify cachexia biomarkers is warranted to better understand catabolism change and assess various therapeutic interventions. Among inflammatory proteins, growth differentiation factor-15 (GDF15), an atypical transforming growth factor-beta (TGF-β) superfamily member, emerges as a stress-related hormone. In inflammatory conditions, cardiovascular diseases, and cancer, GDF15 is a biomarker for disease outcome. GDF15 is also implicated in energy homeostasis, body weight regulation, and plays a distinct role in cachexia. The recent discovery of its receptor, glial cell line-derived neurotrophic factor (GDNF) family receptor α-like (GFRAL), sheds light on its metabolic function. Herein, we critically review the mechanisms involving GDF15 in cancer cachexia and discuss therapeutic interventions to improve outcomes in people living with cancer.

## Introduction

Late stages of diseases such as chronic kidney disease, congestive heart failure, AIDS, and cancer are associated with a wasting syndrome called cachexia.^1^-^3^ For patients with cancer, up to one-third of deaths are attributed to this syndrome. The term 'cachexia' comes from the Greek words “kakos” and “hexis,” which means “bad condition”. Cachexia is defined as a metabolic syndrome associated with extreme involuntary wasting of lean body mass (LBM) with or without loss of fat mass^4^-^9^ The international consensus statement on the definition and classification of cancer cachexia in 2011^6^ established criteria for diagnosing cachexia in patients living with cancer. According to the consensus, cancer cachexia is defined as a loss of edema-free body weight of more than five percent over six months or loss of body weight of more than two percent in patients with a BMI of less than 20 kg/m^2^.^5^, ^7^, ^10^

Weight loss and muscle strength are predictors of mortality in patients with AIDS and cancer.^5^, ^11^-^13^ However, in contrast to malnutrition and starvation which are treated with refeeding, cachexia is not corrected by conventional nutritional support and it is associated with loss of appetite (anorexia).^5^, ^14^, ^15^ It is estimated that systemic inflammation, anorexia, and changes in energy metabolism are the main factors in the development of cachexia.^4^, ^14^, ^16^ Inflammation leading to LBM wasting is induced by both tumor and host-derived factors.^7^ However, the underlying pathophysiology of cachexia remains poorly understood.^8^, ^17^ Moreover, cancer-related anorexia and weight loss remain often untreatable and are associated with treatment resistance.^16^ Therefore, a thorough understanding of the underlying mechanism of weight loss and anorexia is crucial in identifying a metabolic marker that can serve as both a prognostic indicator and a potential therapeutic target in cachexia.^14^, ^16^

## Cachexia in Cancer: As a Late Stage Feature

For cancer patients, cachexia represents a life-threatening condition. Almost eighty percent of advanced cancer patients are affected by involuntary wasting, which is inversely related to handgrip strength, high toxicities of anti-cancer drugs, quality of life, and survival.^7^, ^11^, ^14^, ^16^ For many years scientists and physicians focused efforts on cancer itself, as cachexia was considered a direct consequence of tumor progression. However, cachexia emerges as a distinct condition driven by the combination of inflammation and anorexia creating an overzealous metabolic imbalance in the host.^7^, ^8^, ^14^, ^18^ The key mechanism underlying cachexia is the increased catabolism of muscle protein combined with dampened protein synthesis, leading to LBM loss.^18^

Since cachexia preferentially causes loss of LBM more than FM, there is a need for a biomarker for the early diagnosis of cachexia, especially the atrophy in skeletal muscle.^6^, ^14^ Despite several well-designed interventional clinical trials, cachexia cannot be reversed by conventional nutritional support, appetite stimulant, and recombinant growth hormone.^19^ Specifically, the loss of muscle mass is recognized as an independent predictor of mortality and a major contributor to functional impairment.

In recent years, several markers have been reported to be involved in skeletal muscle atrophy in cancer cachexia.^8^ These noteworthy mediators include the members of the TGFβ family such as Activin (Act) A,^20^, ^21^ growth differentiation factor-11 (GDF11),^22^, ^23^ GDF15,^16^, ^24^, and Myostatin (Mstn).^14^, ^25^-^27^ In 2010, Zhou *et al*. demonstrated reversal of cancer cachexia by injecting anti-bodies against Act A and Mstn in mice model leading to prolong survival.^20^ Recently, a unique role of the inflammatory growth factor GDF15 has been highlighted in many studies using animal and human models of cancer cachexia.^7^, ^8^, ^28^ A study by Lerner *et al*., demonstrated that GDF15 inhibitors reversed the loss of LBM and FM in mice.^8^ These animal studies showed the contribution of inflammatory mediators involved in cachexia.

## Cachexia: A Metabolic Mutiny against Host

In 2005, Fouladiun *et al.* found that cancer-related weight loss was linked to changes in the diet and inflammatory cytokines.^5^, ^29^ Later, several groups reported on the secretion of cachectic factors by the tumor stroma and adjacent immune cells, which were associated with loss of both LBM and FM.^6^, ^7^, ^15^ Lipid metabolism was also affected as increased lipolysis and reduced lipogenesis in the adipocytes were observed during cachexia.^30^ Pro-inflammatory cytokines such as IL-1, IL-6, IL-8, TNFα, and IFNγ acted directly or indirectly on the appetite centers of the central nervous system causing anorexia.^8^, ^14^, ^16^, ^31^, ^32^ Moreover, the use of anti-TNFα or anti-IL-6 monoclonal antibodies did not influence survival or weight loss.^8^, ^19^, ^33^, ^34^. This suggests the need for a better understanding of the pathogenesis of cachexia that can serve both as a prognostic indicator and as a therapeutic target for reversing weight loss.^14^ In humans, however, several clinical trials are ongoing to modulate plasma cytokines to revert cancer cachexia using NGM120 agent and GDF15 with induction chemotherapy.^14^, ^33^ **(Table 1).**

## Contribution of Gdf15 to Anorexia and Cachexia

Almost two decades ago GDF15 was identified as a member of the TGF-β superfamily.^7^, ^35^-^37^ Although it has been given different names, including macrophage inhibitory cytokine 1 (MIC-1), NSAID activated gene 1 (NAG-1), placental bone morphogenetic protein (PLAB), placental transforming growth factor-beta (PTGFB), prostate derived factor (PDF), following Strelau* et al*.'s recommendation in 2000, the protein was coined growth differentiation factor 15 (GDF15).^35^, ^38^, ^39^ Outside its role in the placenta, GDF15 is a stress-induced hormone that is released by many tissues including vascular smooth muscle, cardiac and endothelial cells, macrophages, and adipocytes in response to diverse pro-inflammatory conditions such as obesity, insulin resistance, heart failure, and cancer.^7^, ^40^, ^41^ In relatively healthy individuals, the levels of GDF15 rise with age, intense exercise, obesity, smoking, pregnancy, and use of metformin.^42^-^45^ GDF15 is expressed in higher concentrations in the placenta and prostate and is released in homeostatic conditions in smaller amounts by other tissues such as the liver, colon, stomach, heart, lungs, bladder, and kidneys. Conversely, higher levels are released by tissue damage and stress creating a peripheral non-homeostatic regulation of neural circuitry.^7^, ^39^, ^41^, ^43^, ^46^, ^47^ Plasma levels usually range from 100 to 1200 pg/ml, and a biphasic pattern in serum GDF15 levels was observed with a peak around midnight.^43^, ^48^, ^49^

Several studies demonstrated the overexpression of GDF15 in many advanced cancers such as prostate, urothelial, breast, gastric, colorectal, and esophagus.^24^, ^28^, ^47^, ^50^, ^51^ In advanced cancers, the circulating levels of GDF15 might surge up to 10,000-100,000 pg/mL.^24^, ^48^, ^52^ Furthermore, a negative association between the increased plasma levels of GDF15 and survival was found in many cancers.^28^, ^50^, ^51^, ^53^-^57^ In 2007, Johnen *et al*. injected GDF15 subcutaneously into mice which caused a rapid decrease in food intake and hence, a decline in weight.^24^, ^28^ Moreover, Macia *et al*. in 2012 further demonstrated that the overexpression of GDF15 in tumors led to the inhibition of food intake in an animal model which subsequently causes loss of both LBM and FM.^58^ Circulating levels of GDF15 and subsequent weight loss were associated with the stage and extent of disease progression and with all-cause mortality.^58^

GDF15 may have a context-dependent role in cancers: an antitumorigenic role of GDF15 was observed in early cancer while an association of GDF15 with the induction of tumor growth was observed in advanced cancer depending on a tumor type and its micro-environment.^28^, ^35^, ^46^, ^47^ Early in the disease, the overexpression of GDF15 in cancer cells is speculated to be involved in the induction of apoptosis, and hence, anti-tumorigenicity.^28^ Studies demonstrated a chemoprotective role of GDF15 in colon cancer and colonic polyps in mice models.^59^-^61^ Similarly, one study in 2002 showed the anti-angiogenic properties of GDF15 *in-vivo* and* in-vitro studies* which were responsible for its anti-tumorigenic effect.^62^ Later it was found that elevated levels of GDF15 are implicated in the causation of both chronic inflammation and tumorigenicity.^28^ Brown *et al*. demonstrated a strong association of elevated plasma GDF15 levels with metastasis in prostate cancer.^51^ Another study performed on 41 patients with testicular carcinoma, revealed the association of elevated levels of GDF15 with endothelial damage.^63^ Globally, GDF15 in cancer seems to plays an anti-tumorigenic response to limit tumor growth at the early stages of cancer development while in the later stages, tumors may use GDF15 to escape immune surveillance to expand.^46^

In one animal study, GDF15 was identified as the only biomarker for the loss of skeletal muscle and weight in cancer.^16^ Both GDF15 and to a lesser extent IL-8 were inversely linked to survival.^16^, ^64^ Interestingly, weight loss was reversed by the administration of monoclonal antibodies against GDF15 and restored skeletal muscle and FM in several cachectic animal models.^8^, ^15^, ^16^, ^28^, ^46^ Additionally, antibody-treated mice exhibited increased food intake, better locomotor function, energy expenditure, and resulted in de novo synthesis of muscle and adipose tissue.^8^ A combination of tivozanib, an oral VEGF receptor tyrosine kinase inhibitor, and a GDF15 inhibitor further resulted in increased survival owing to the reversal of weight loss as compared to the control.^8^ Such study findings indicate that GDF15 is much more than a biomarker of anorexia as it induces a distinctive “metabolic signature” characterized by enhanced lipid oxidation and muscle atrophy, leading to cachexia.^65^

## Gdf15 and the Identification of its Neuroreceptor Gfral

**Figure 1:**
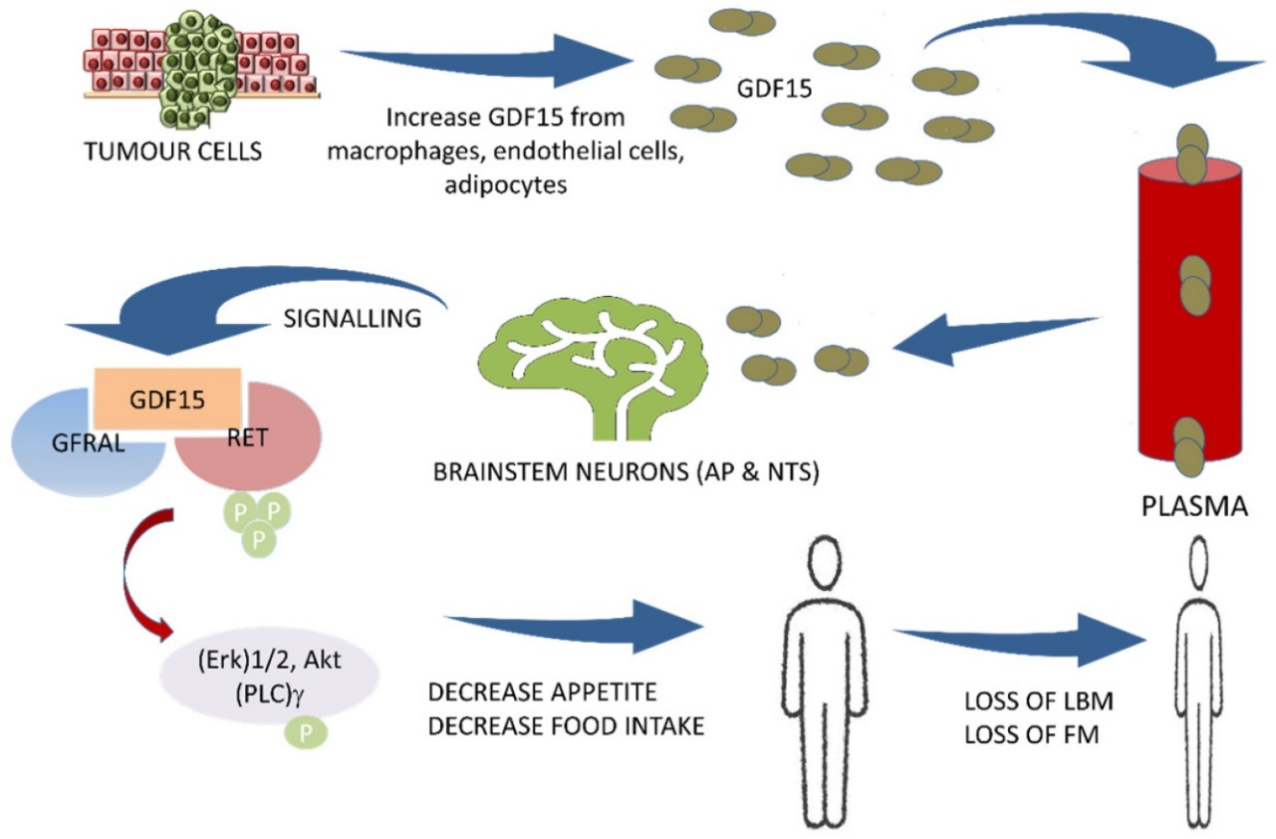
The central pathway of cancer cachexia involving GDF15 and GFRAL with RET proto-oncogene as a coreceptor. AP: area postrema; NTS: nucleus of solitary tract; GDF15: growth differentiation factor15; GFRAL: GDNF family receptor alpha-like; RET: rearranged during transfection proto-oncogene, a co-receptor to GFRAL; Erk: extracellular-signal-regulated kinase; Akt: Ak strain transforming; (PLC)γ: phospholipase C-gamma; LBM: Lean body mass; FM: fat mass

In 2017, the GDF15 receptor was identified as the glial cell-derived neurotrophic factor (GDNF) family receptor alpha-like (GFRAL) expressed in the brainstem.^3^, ^40^, ^66^, ^67^ The GDF15/GFRAL pathway has emerged as a key regulator of energy balance and anorexia. By binding to its neuroreceptor GFRAL, GDF15 induces a dramatic reduction in appetite, LBM, and FM.^16^ Furthermore, it was found that recombinant GDF15 suppresses food intake, regulates energy expenditure, and enhances weight loss through interactions with its unique brainstem-restricted neurotropic receptor, GFRAL.^7^, ^36^, ^40^, ^41^, ^44^, ^48^ GDF15 acts specifically on the appetite center in the hypothalamus and brainstem mainly the area postrema (AP) and nucleus of the solitary tract (NTS) and controls the appetite centrally.^3^, ^18^, ^40^, ^68^ The complex of GDF15 and GFRAL with its co-receptor the proto-oncogene RET (rearranged during transfection) causes activation with subsequent phosphorylation of intracellular signaling molecules, (Erk)1/2: extracellular signal-regulated kinase, Akt: Ak strain tranforming, and (PLC)γ: phospholipase C-gamma.^18^, ^40^ (Figure 1) It is well established that GDF15 regulates food intake, energy expenditure, and body weight in response to metabolic and toxin-induced stresses in cancer. Furthermore, the mechanism regulating the GDF15 metabolic effect has recently been identified. In 2015, Tsai *et al*. showed an absence of a postprandial increase in GDF15 serum levels in healthy participants suggesting that it is unlikely that this hormone acts as a 'satiety factor'.^47^ O'Rahilly *et al*., in 2017 showed that GDF15 serum levels induced a minimal change in response to caloric surpluses or deficits in both mice and humans, differentiating its action from intestinally derived satiety hormones and leptin. Furthermore, the same group showed that GDF15 expression was regulated by the tissue stress response in mice, and its administration triggered conditioned taste aversion suggesting that GDF15 induces an aversive response to nutritional stress.^69^ In addition, Patel *et al*. in 2019, also demonstrated that GDF15 administration triggered conditioned taste aversion via GFRAL, while Borner *et al.* showed that GDF15 Induces anorexia through nausea and emesis in the mouse model. Globally, these findings indicate that GDF15 does not act as an 'anti-hunger' hormone or has any 'satiety effect' but induces an aversive response to nutritional stress. 44, 70 GDF-15 and GFRAL signaling pathways are thus suggested to be an important potential therapeutic target for both cancer cachexia and obesity.^7^, ^16^, ^42^, ^46^, ^54^, ^71^ Thus, both GDF15 and GFRAL signaling through the tyrosine kinase receptor RET^18^, ^46^ are under investigation in several clinical trials.^43^, ^46^ (Table 1)

## Gdf15 Induces Anorexia, Lipid Oxidation, and Muscle Atrophy via Neuroendocrine Axis

It was established in the 1980s that the β adrenergic stimulation causes increase lipolysis and inhibit the activity of the lipoprotein lipase resulting in increased plasma triglycerides.^72^ In 2019, Luan *et al*. identified novel characteristics of GDF15 in disease tolerance in the context of infection. This group showed in a mouse model that GDF15 is induced in inflammatory diseases through the central induction of metabolic adaptation and contribute to a protective effect in organ damage. This inflammation-induced tolerance effect is achieved by metabolic reprogramming and the production of triglycerides via hepatic sympathetic outflow, hence preventing cardiac damage after an LPS endotoxemic challenge.^73^ Such protective activity on tissue after the infection is achieved by central activation via the GFRAL receptor and production of norepinephrine with subsequent stimulation of the liver to produce and release triglycerides.^47^, ^73^, ^74^ These findings are supported by previous reports on the cardioprotective role of GDF15 in the context of inflammation and infection.^75^, ^76^ However, the relevance of the GDF15 adrenergic pathway contribution in cancer cachexia, which is also characterized by increased lipolysis, was not determined.^30^, ^73^

In August 2020, Suriben et al. were the first to link the GDF15/GRFAL sympathetic pathway with the hepatic lipid oxidation in a cancer cachexia mouse model.^65^ The injection of a monoclonal antibody antagonist that targets GFRAL and inhibit Ret proto-oncogene (RET) signaling complex in brainstem neurons (area postrema: AP; and nucleus of solitary tract: NTS) reversed excessive lipid oxidation in tumor-bearing mice and prevents cancer cachexia.^65^ Furthermore, to unveil the mechanism of action of GDF15, the same group showed that activation of the GFRAL-RET pathway induces the expression of genes involved in lipid metabolism in adipose tissues. They also showed that chemical peripheral sympathectomy and the loss of adipose triglyceride lipase protect mice from GDF15-induced weight loss in the context of cancer.^65^ This ground-breaking observation demonstrated the role of the peripheral sympathetic axis by which GDF15 elicits a lipolytic response in adipose tissue independently of anorexia, contributing to the reduction in both, adipose and muscle mass in tumor-bearing mice.

## Challenges of Blocking Gdf15/Gfral Pathway: Consideration for Cachexia and Cancer Progression

Despite the breakthrough in inhibiting the GDF15/GFRAL pathway for the treatment of cachexia in the mouse model, several issues concerning interplay between host and tumor must be addressed for cachectic cancer patients.^65^

### Effect on cachexia

For most patients with metastatic cancers, the cause of death is due to cachexia more than the direct effect of tumor bulk and organ failure. Based on the absence of any therapy for improving cachexia and on similar mechanistic evidence in the animal model and human, clinical trials testing anti-GDF15/GFRAL treatments appear promising, specifically in patients with elevated circulating GDF15 levels.^65^ Considering the limited clinical consequence of GDF15 knock-out mice, acceptable tolerability can be expected. Still, due to the limited sequence homology between mice and human GDF15, studies in humans may not fully replicate those from mouse models.

### Effect on cancer progression

Beyond its cachectic effect, GDF15 has been also implicated in tumor cell apoptosis and the development of metastasis. In addition, GDF15 can also modulate the tumor microenvironment, innate immune surveillance, and response to immunetherapy.^77^ As the tissue distribution of GFRAL is limited to the hindbrain, direct effects of GDF15 on cancer or microenvironment immune cells may be mediated by yet unidentified GFRAL-independent signaling pathways.^47^ As such, consequences of direct inhibition of GDF15 on the tumor itself may be more difficult to predict. Encouragingly, recent study findings suggest that blockade of GDF15 has the potential to reverse anorexia while improving response immunotherapies.^47^

Globally, considering both host and cancer factors, only the completion of clinical trials using an inhibitor of the GDF15/GFRAL pathway will provide clinical evidence on the merit of reversing this “metabolic signature” to improve the lives of those living with advanced cancer.^78^-^81^

## Conclusion

GDF15 plasma level correlates with tumor progression and has been considered as a tumor biomarker.^47^, ^55^, ^82^-^86^ Functionally, GDF15 is now considered as the main actor of cachexia in cancer signaling through its neuroreceptor GFRAL. However, via an adrenergic pathway GDF15 can stimulate hepatic triglyceride release to protect tissues from inflammatory stress. Overall, reports of GDF15-dependent effects in cancer are context-dependent and mainly studied in animal models. Therefore, results from ongoing clinical trials in human models inhibiting the GDF15/GFRAL pathway will shed light on patients with cancer cachexia. Due to the complex contribution of cancer and host factors, combination therapy including GDF15 blockade along with chemotherapy/immunotherapy will likely be needed to reverse cachexia metabolic signature, which in turn will improve the lives of persons living with cancer.

## Figures and Tables

**Table 1:** Ongoing clinical trials in patients with cancer cachexia.

Study	Study type	Study title	Phase	Intervention/treatment	Time frame	Participants	Location
NCT04068896	RCT	A Phase 1a/b dose escalation study followed by expansion cohorts of NGM120, a GFRAL antagonist monoclonal antibody blocking GDF15 signaling, in subjects with advanced solid tumors receiving combination therapy	Phase 1a/b dose-escalation study	NGM120 x 6 doses of Subcutaneous injection	19 weeks	n=90	United States
NCT02285530	RCT	GDF15 based TPF induction chemotherapy for oral squamous cell carcinoma patients at T3/T4cN0M0 stage	Phase 2	TPF induction chemotherapy docetaxel:75mg/m2; cisplatin:75 mg/m2; 5-Fu:750 mg/m2/day (2 cycles)	2 years	n=72	China
NCT03743051	RCT, double-blind, placebo-controlled	Efficacy and safety of Anamorelin HCl in adult patients with advanced Non-Small Cell Lung Cancer	Phase 3	Anamorelin Hydrochloride 100mg oral tablets Drug: Placebo Oral Tablet	9 weeks from baseline	n=316	United States
NCT03283488	RCT, double-blind	Mirtazapine vs. Megestrol	Phase 3	Mirtazapine 15mg oral tablet vs. Megestrol 160mg oral tablet	8 weeks	n=40	Brazil
NCT01614990	RCT Pilot study	A pilot clinical trial of Macimorelin	Phase 2	Macimorelin (1 mg/kg) daily	7 days	n=8	United States
NCT03207724	Interventional Clinical Trial Single Group Assignment	A Phase I study of Onivyde and 5-FU in combination with Xilonix for advanced pancreatic cancer	Phase 1	Xilonix plus Onivyde and 5FU (IV)	28 days, 6 months, 12 months from baseline	n=16	United States
NCT03263520	RCT	Nandrolone on the treatment of malnutrition induced by cancer	Phase 1	Nandrolone Decanoate 25 or 50mg (M), 25mg (F) IM and Dexamethasone 4mg for M&F	30 days	n=60	Brazil
NCT03254173	RCT	Mirtazapine for treatment of cancer patients	Phase 2 Phase 3	Mirtazapine 30 mg oral tablets Placebo oral tablets	8 weeks	n=120	Egypt
NCT02359123	Interventional Clinical Trial- Pilot Study	Cannabidiol capsules as a treatment to improve cancer-related cachexia and anorexia syndrome in advanced cancer patients	Phase 1	Cannabis extract in an oil formulation capsules 5mg	3 months	n=24	Israel

## Abbreviations

GDF15: growth differentiation factor 15
GFRAL: glial cell line-derived neurotrophic factor (GDNF) family receptor α-like
HIV: human immunodeficiency virus
AIDS: acquired immune deficiency syndrome
LBM: lean body mass
FM: fat mass
TGFβ: transforming growth factor-beta
Act A: activin A
GDF11: growth differentiation factor 11
Mstn: myostatin
IL: interleukin
TNFα: tumor necrosis factor-alpha
IFNγ: interferon-gamma
BMI: basal mass index
MIC-1: macrophage inhibitory cytokine 1
NAG-1: NSAID activated gene 1
PLAB: placental bone morphogenetic protein
PTGFB: placental transforming growth factor-beta
PDF: prostate derived factor
VEGF: vascular endothelial growth factor
AP: area postrema
NTS: nucleus of solitary nucleus
RET: rearranged during transfection
LPS: lipopolysaccharide
Erk: extracellular signal-regulated kinase
Akt: Ak strain transforming
(PLC)γ: phospholipase C-gamma
